# Triaqua­(2,2′-bipyridine)(5-nitro­isophthal­ato-κ*O*)nickel(II) monohydrate

**DOI:** 10.1107/S1600536808038233

**Published:** 2008-12-10

**Authors:** Ying Liu, Qingpeng He, Xianxi Zhang, Zechun Xue, Chunyan Lv

**Affiliations:** a College of Chemistry and Chemical Engineering, Liaocheng University, Liaocheng 252059, People’s Republic of China

## Abstract

In the title compound, [Ni(C_8_H_3_NO_6_)(C_10_H_8_N_2_)(H_2_O)_3_]·H_2_O, the Ni^II^ cation is six-coordinated by a chelating 2,2′-bipyridine ligand, one carboxyl­ate O atom from a 5-nitro­isophthalate dianion and three water mol­ecules, with a slightly distorted *cis*-NiN_2_O_4_ octa­hedral geometry. The neutral complex is isolated, in contrast to coordination polymers formed by Mn^II^, Co^II^ and Cu^II^ with the same ligand set, but forms an extensive network of O—H⋯O hydrogen bonds between the coordinated and uncoordinated water mol­ecules and carboxyl­ate groups of the 5-nitro­isophthalate ions.

## Related literature

For the related coordination polymers containing Co^II^, Mn^II^ and Cu^II^, see: Xiao *et al.* (2005 ▶); Xie *et al.* (2005 ▶, 2006 ▶), respectively. For background, see: Kim *et al.* (2001 ▶).
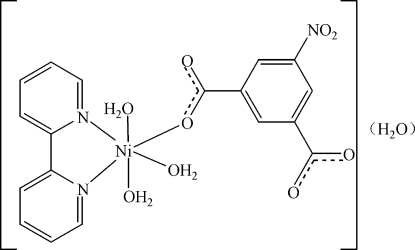


## Experimental

### 

#### Crystal data

[Ni(C_8_H_3_NO_6_)(C_10_H_8_N_2_)(H_2_O)_3_]·H_2_O
*M* *_r_* = 496.05
Triclinic, 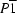

*a* = 7.4867 (10) Å
*b* = 10.717 (3) Å
*c* = 12.773 (2) Å
α = 89.798 (10)°
β = 87.89 (2)°
γ = 74.675 (10)°
*V* = 987.7 (3) Å^3^
*Z* = 2
Mo *K*α radiation
μ = 1.05 mm^−1^
*T* = 293 (2) K
0.12 × 0.10 × 0.08 mm

#### Data collection

Bruker APEXII CCD diffractometer
Absorption correction: multi-scan (*SADABS*; Bruker, 2001 ▶) *T*_min_ = 0.885, *T*_max_ = 0.921
5649 measured reflections
3823 independent reflections
3271 reflections with *I* > 2σ(*I*)
*R*_int_ = 0.016

#### Refinement

*R*[*F* ^2^ > 2σ(*F* ^2^)] = 0.043
*wR*(*F* ^2^) = 0.122
*S* = 1.01
3823 reflections
314 parameters
12 restraints
H atoms treated by a mixture of independent and constrained refinement
Δρ_max_ = 0.99 e Å^−3^
Δρ_min_ = −0.73 e Å^−3^

### 

Data collection: *APEX2* (Bruker, 2004 ▶); cell refinement: *SAINT-Plus* (Bruker, 2001 ▶); data reduction: *SAINT-Plus*; program(s) used to solve structure: *SHELXS97* (Sheldrick, 2008 ▶); program(s) used to refine structure: *SHELXL97* (Sheldrick, 2008 ▶); molecular graphics: *SHELXTL* (Sheldrick, 2008 ▶); software used to prepare material for publication: *SHELXTL*.

## Supplementary Material

Crystal structure: contains datablocks global, I. DOI: 10.1107/S1600536808038233/hb2841sup1.cif

Structure factors: contains datablocks I. DOI: 10.1107/S1600536808038233/hb2841Isup2.hkl

Additional supplementary materials:  crystallographic information; 3D view; checkCIF report

## Figures and Tables

**Table 1:** Selected bond lengths (Å)

Ni1—O1	2.051 (2)
Ni1—O2*W*	2.079 (2)
Ni1—O1*W*	2.088 (2)
Ni1—O3*W*	2.142 (2)
Ni1—N2	2.097 (2)
Ni1—N3	2.100 (2)

**Table 2:** Hydrogen-bond geometry (Å, °)

*D*—H⋯*A*	*D*—H	H⋯*A*	*D*⋯*A*	*D*—H⋯*A*
O1*W*—H1*W*⋯O2	0.81 (3)	2.00 (3)	2.727 (3)	150 (5)
O1*W*—H2*W*⋯O4^i^	0.82 (4)	1.96 (5)	2.715 (3)	154 (4)
O2*W*—H3*W*⋯O4*W*^ii^	0.82 (3)	1.82 (4)	2.614 (4)	166 (4)
O2*W*—H4*W*⋯O4^iii^	0.82 (4)	1.96 (4)	2.757 (3)	164 (4)
O3*W*—H5*W*⋯O4*W*^iv^	0.82 (4)	2.42 (2)	3.185 (7)	156 (5)
O3*W*—H6*W*⋯O4^v^	0.82 (3)	1.97 (2)	2.735 (3)	155 (4)
O4*W*—H7*W*⋯O2^vi^	0.81 (4)	1.91 (2)	2.697 (4)	161 (5)
O4*W*—H8*W*⋯O3^vii^	0.81 (3)	1.98 (4)	2.639 (4)	138 (5)

Symmetry codes: (i) 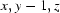; (ii) 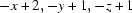; (iii) 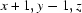; (iv) 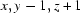; (v) 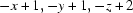; (vi) 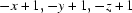; (vii) 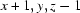.

**Fig. 1.**
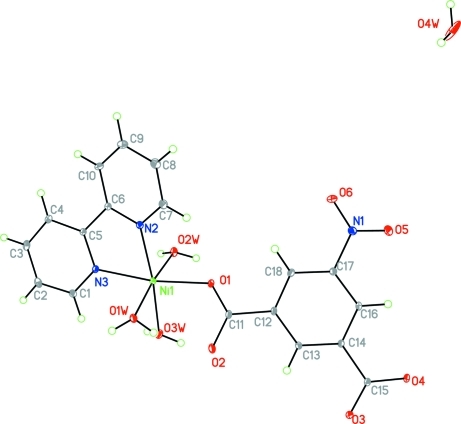
A view of the molecular structure of (I), showing 30% probability displacement ellipsoids for the non-hydrogen atoms.

## supplementary crystallographic information

### Comment

In recent years, carboxylic acids have been widely used in materials science as polydentate ligands which can coordinate to transition-metal or rare-earth cations to yield complexes with interesting or useful properties. For example, Kim *et al.* (2001) have focused on the syntheses of transition-metal complexes containing benzene carboxylate and rigid aromatic pyridine ligands in order to study their electronic conductivity and magnetic properties. The importance of transition-metal dicarboxylate complexes motivated us to pursue synthetic strategies for these compounds, using 5-nitroisophthalic acid as a polydentate ligand and we now report the synthesis and structure of the title compound, (I) (Fig. 1).

The Ni^II^ cation in (I) is hexa-coordinated by a chelating 2,2'-bipyridine ligand, one carboxylate O atom from a 5-nitroisophthalate dianion and three water molecules, with a slightly distorted cis-NiN_2_O_4_ octahedral geometry (Table 1). The neutral complex is isolated, in contrast to coordination polymers formed by Mn^II^, Co^II^ and Cu^II^ with the same ligand set, but forms an extensive network of O—H···O hydrogen bonds (Table 2) between the coordinated and uncoordinated water molecules and carboxylate groups of the 5-nitroisophthalate ions.

### Experimental

A mixture of nickel dichloride (0.5 mmol), 2,2'-bipyridine (0.5 mmol), and 5-nitroisophthalic acid (0.5 mmol) in H_2_O (8 ml) and ethanol (8 ml) was sealed in a 25-ml Teflon-lined stainless steel autoclave and heated to 413 K for three days.  Green blocks of (I) were obtained after cooling to room temperature with a yield of 27%.

### Refinement

The H atoms of the water molecule were located from difference density maps and were refined with distance restraints of H···H = 1.38 (2) Å, O—) = 0.88 (2) Å, and with a fixed *U*_iso_ of 0.80 Å^2^. All other H atoms were placed in calculated positions with a C—H bond distance of 0.93 Å and *U*_iso_(H) = 1.2*U*_eq_ of the carrier atom.

### Crystal data

**Table:**

[Ni(C_8_H_3_NO_6_)(C_10_H_8_N_2_)(H_2_O)_3_]·H_2_O	*Z* = 2
*M**_r_* = 496.05	*F*(000) = 512
Triclinic, *P*1	*D*_x_ = 1.668 Mg m^−^^3^
Hall symbol: -P 1	Mo *K*α radiation, λ = 0.71073 Å
*a* = 7.4867 (10) Å	Cell parameters from 3823 reflections
*b* = 10.717 (3) Å	θ = 1.6–26.0°
*c* = 12.773 (2) Å	µ = 1.05 mm^−^^1^
α = 89.798 (10)°	*T* = 293 K
β = 87.89 (2)°	Block, green
γ = 74.675 (10)°	0.12 × 0.10 × 0.08 mm
*V* = 987.7 (3) Å^3^	

### Data collection

**Table:**

Bruker APEXII CCD diffractometer	3823 independent reflections
Radiation source: fine-focus sealed tube	3271 reflections with *I* > 2σ(*I*)
graphite	*R*_int_ = 0.016
ω scans	θ_max_ = 26.0°, θ_min_ = 1.6°
Absorption correction: multi-scan (*SADABS*; Bruker, 2001)	*h* = −8→9
*T*_min_ = 0.885, *T*_max_ = 0.921	*k* = −13→11
5649 measured reflections	*l* = −15→15

### Refinement

**Table:**

Refinement on *F*^2^	Primary atom site location: structure-invariant direct methods
Least-squares matrix: full	Secondary atom site location: difference Fourier map
*R*[*F*^2^ > 2σ(*F*^2^)] = 0.043	Hydrogen site location: difmap and geom
*wR*(*F*^2^) = 0.122	H atoms treated by a mixture of independent and constrained refinement
*S* = 1.00	*w* = 1/[σ^2^(*F*_o_^2^) + (0.071*P*)^2^ + 0.9983*P*] where *P* = (*F*_o_^2^ + 2*F*_c_^2^)/3
3823 reflections	(Δ/σ)_max_ < 0.001
314 parameters	Δρ_max_ = 0.99 e Å^−^^3^
12 restraints	Δρ_min_ = −0.73 e Å^−^^3^

### Special details

**Table:**

Geometry. All e.s.d.'s (except the e.s.d. in the dihedral angle between two l.s. planes) are estimated using the full covariance matrix. The cell e.s.d.'s are taken into account individually in the estimation of e.s.d.'s in distances, angles and torsion angles; correlations between e.s.d.'s in cell parameters are only used when they are defined by crystal symmetry. An approximate (isotropic) treatment of cell e.s.d.'s is used for estimating e.s.d.'s involving l.s. planes.
Refinement. Refinement of *F*^2^ against ALL reflections. The weighted *R*-factor *wR* and goodness of fit *S* are based on *F*^2^, conventional *R*-factors *R* are based on *F*, with *F* set to zero for negative *F*^2^. The threshold expression of *F*^2^ > σ(*F*^2^) is used only for calculating *R*-factors(gt) *etc*. and is not relevant to the choice of reflections for refinement. *R*-factors based on *F*^2^ are statistically about twice as large as those based on *F*, and *R*- factors based on ALL data will be even larger.

### Fractional atomic coordinates and isotropic or equivalent isotropic displacement parameters (Å^2^)

**Table:**

	*x*	*y*	*z*	*U*_iso_*/*U*_eq_	
Ni1	0.72908 (5)	−0.02881 (3)	0.78571 (3)	0.02430 (16)	
C1	0.8232 (5)	−0.3096 (3)	0.8659 (2)	0.0303 (7)	
H1	0.7701	−0.2738	0.9296	0.036*	
C2	0.8972 (5)	−0.4404 (3)	0.8591 (3)	0.0371 (8)	
H2	0.8903	−0.4931	0.9163	0.044*	
C3	0.9816 (5)	−0.4922 (3)	0.7667 (3)	0.0385 (8)	
H3	1.0368	−0.5807	0.7612	0.046*	
C4	0.9848 (5)	−0.4139 (3)	0.6820 (3)	0.0321 (7)	
H4	1.0424	−0.4480	0.6187	0.038*	
C5	0.9009 (4)	−0.2836 (3)	0.6926 (2)	0.0227 (6)	
C6	0.8764 (4)	−0.1940 (3)	0.6036 (2)	0.0254 (6)	
C7	0.7338 (5)	0.0090 (3)	0.5475 (3)	0.0360 (8)	
H7	0.6633	0.0927	0.5628	0.043*	
C8	0.7884 (6)	−0.0224 (4)	0.4454 (3)	0.0476 (9)	
H8	0.7524	0.0376	0.3922	0.057*	
C9	0.8975 (7)	−0.1445 (4)	0.4233 (3)	0.0532 (11)	
H9	0.9406	−0.1680	0.3551	0.064*	
C10	0.9418 (6)	−0.2309 (4)	0.5028 (3)	0.0429 (9)	
H10	1.0157	−0.3141	0.4893	0.052*	
C11	0.5174 (4)	0.2464 (3)	0.8072 (3)	0.0296 (7)	
C12	0.4966 (4)	0.3864 (3)	0.7871 (2)	0.0245 (6)	
C13	0.4091 (4)	0.4774 (3)	0.8608 (2)	0.0244 (6)	
H13	0.3675	0.4506	0.9243	0.029*	
C14	0.3821 (4)	0.6079 (3)	0.8421 (2)	0.0218 (6)	
C15	0.2887 (4)	0.7042 (3)	0.9250 (2)	0.0229 (6)	
C16	0.4381 (4)	0.6470 (3)	0.7464 (2)	0.0242 (6)	
H16	0.4168	0.7345	0.7311	0.029*	
C17	0.5258 (4)	0.5548 (3)	0.6739 (2)	0.0237 (6)	
C18	0.5605 (4)	0.4251 (3)	0.6923 (2)	0.0267 (6)	
H18	0.6250	0.3647	0.6429	0.032*	
H1W	0.394 (6)	0.0433 (19)	0.814 (4)	0.080*	
H2W	0.430 (7)	−0.089 (3)	0.824 (3)	0.080*	
H3W	1.002 (7)	0.0536 (18)	0.815 (4)	0.080*	
H4W	1.071 (6)	−0.077 (3)	0.827 (3)	0.080*	
H5W	0.784 (5)	−0.065 (3)	0.979 (4)	0.080*	
H6W	0.696 (6)	0.062 (2)	0.980 (4)	0.080*	
H7W	0.857 (4)	0.785 (5)	0.129 (4)	0.080*	
H8W	1.005 (6)	0.792 (5)	0.064 (2)	0.080*	
N1	0.5843 (4)	0.5975 (3)	0.5725 (2)	0.0317 (6)	
N2	0.7764 (3)	−0.0739 (2)	0.62575 (19)	0.0254 (5)	
N3	0.8242 (3)	−0.2316 (2)	0.78473 (18)	0.0236 (5)	
O1	0.6531 (3)	0.16736 (19)	0.76279 (17)	0.0296 (5)	
O2	0.3983 (4)	0.2176 (2)	0.8663 (3)	0.0569 (8)	
O3	0.2273 (3)	0.6637 (2)	1.00532 (18)	0.0380 (6)	
O4	0.2779 (3)	0.82113 (19)	0.90681 (17)	0.0294 (5)	
O5	0.5189 (4)	0.7083 (2)	0.54771 (19)	0.0495 (7)	
O6	0.6974 (4)	0.5196 (2)	0.51729 (18)	0.0413 (6)	
O1W	0.4496 (3)	−0.0279 (2)	0.79179 (19)	0.0316 (5)	
O2W	1.0016 (3)	−0.0185 (2)	0.79477 (19)	0.0318 (5)	
O3W	0.7027 (4)	−0.0088 (2)	0.95279 (18)	0.0391 (6)	
O4W	0.9500 (5)	0.8091 (4)	0.1199 (5)	0.115 (2)	

### Atomic displacement parameters (Å^2^)

**Table:**

	*U*^11^	*U*^22^	*U*^33^	*U*^12^	*U*^13^	*U*^23^
Ni1	0.0280 (2)	0.0187 (2)	0.0255 (2)	−0.00530 (16)	0.00210 (15)	−0.00138 (14)
C1	0.0380 (18)	0.0296 (16)	0.0247 (15)	−0.0118 (14)	−0.0005 (13)	0.0040 (12)
C2	0.043 (2)	0.0311 (18)	0.0399 (19)	−0.0143 (15)	−0.0094 (15)	0.0133 (14)
C3	0.043 (2)	0.0190 (15)	0.051 (2)	−0.0035 (14)	−0.0063 (16)	0.0027 (14)
C4	0.0348 (17)	0.0220 (15)	0.0365 (17)	−0.0031 (13)	0.0035 (14)	−0.0048 (13)
C5	0.0252 (15)	0.0196 (14)	0.0234 (14)	−0.0059 (11)	0.0000 (11)	−0.0005 (11)
C6	0.0297 (16)	0.0206 (14)	0.0260 (15)	−0.0072 (12)	0.0024 (12)	−0.0012 (11)
C7	0.046 (2)	0.0288 (17)	0.0308 (17)	−0.0059 (15)	−0.0046 (14)	0.0077 (13)
C8	0.072 (3)	0.043 (2)	0.0282 (18)	−0.016 (2)	−0.0062 (17)	0.0108 (15)
C9	0.086 (3)	0.051 (2)	0.0237 (17)	−0.023 (2)	0.0098 (18)	−0.0020 (16)
C10	0.064 (2)	0.0338 (18)	0.0284 (17)	−0.0100 (17)	0.0155 (16)	−0.0061 (14)
C11	0.0266 (16)	0.0163 (14)	0.0451 (18)	−0.0048 (12)	0.0056 (14)	−0.0013 (13)
C12	0.0228 (14)	0.0147 (13)	0.0349 (16)	−0.0032 (11)	0.0015 (12)	0.0003 (11)
C13	0.0242 (15)	0.0192 (14)	0.0292 (15)	−0.0053 (11)	0.0028 (12)	0.0023 (11)
C14	0.0218 (14)	0.0180 (14)	0.0256 (14)	−0.0053 (11)	−0.0004 (11)	0.0000 (11)
C15	0.0230 (14)	0.0168 (13)	0.0276 (15)	−0.0030 (11)	−0.0022 (11)	0.0005 (11)
C16	0.0289 (15)	0.0158 (13)	0.0284 (15)	−0.0069 (11)	−0.0034 (12)	0.0014 (11)
C17	0.0274 (15)	0.0230 (14)	0.0210 (14)	−0.0069 (12)	−0.0013 (11)	−0.0001 (11)
C18	0.0287 (15)	0.0210 (14)	0.0296 (15)	−0.0053 (12)	0.0009 (12)	−0.0049 (12)
N1	0.0414 (16)	0.0322 (15)	0.0241 (13)	−0.0147 (12)	−0.0001 (11)	−0.0007 (11)
N2	0.0306 (13)	0.0224 (12)	0.0237 (12)	−0.0079 (10)	−0.0006 (10)	0.0018 (10)
N3	0.0288 (13)	0.0186 (12)	0.0234 (12)	−0.0066 (10)	−0.0006 (10)	−0.0007 (9)
O1	0.0319 (12)	0.0136 (10)	0.0401 (12)	−0.0018 (8)	0.0103 (10)	−0.0012 (8)
O2	0.0483 (16)	0.0202 (12)	0.100 (2)	−0.0103 (11)	0.0408 (16)	−0.0044 (13)
O3	0.0522 (15)	0.0225 (11)	0.0353 (12)	−0.0055 (10)	0.0178 (11)	0.0009 (9)
O4	0.0399 (12)	0.0151 (10)	0.0314 (11)	−0.0049 (9)	0.0044 (9)	−0.0014 (8)
O5	0.080 (2)	0.0305 (14)	0.0337 (13)	−0.0095 (13)	0.0069 (13)	0.0105 (10)
O6	0.0484 (15)	0.0429 (14)	0.0293 (12)	−0.0085 (12)	0.0131 (11)	−0.0061 (10)
O1W	0.0281 (12)	0.0221 (11)	0.0443 (13)	−0.0073 (9)	0.0061 (10)	0.0013 (10)
O2W	0.0297 (12)	0.0237 (11)	0.0424 (13)	−0.0070 (9)	−0.0075 (10)	0.0023 (10)
O3W	0.0660 (18)	0.0267 (12)	0.0248 (11)	−0.0132 (12)	0.0003 (11)	−0.0043 (9)
O4W	0.0426 (19)	0.079 (3)	0.227 (6)	−0.0297 (18)	0.054 (3)	−0.107 (3)

### Geometric parameters (Å, °)

**Table:**

Ni1—O1	2.051 (2)	C11—O2	1.246 (4)
Ni1—O2W	2.079 (2)	C11—O1	1.256 (4)
Ni1—O1W	2.088 (2)	C11—C12	1.490 (4)
Ni1—O3W	2.142 (2)	C12—C13	1.375 (4)
Ni1—N2	2.097 (2)	C12—C18	1.387 (4)
Ni1—N3	2.100 (2)	C13—C14	1.380 (4)
C1—N3	1.331 (4)	C13—H13	0.9300
C1—C2	1.365 (5)	C14—C16	1.377 (4)
C1—H1	0.9300	C14—C15	1.499 (4)
C2—C3	1.366 (5)	C15—O3	1.232 (4)
C2—H2	0.9300	C15—O4	1.256 (3)
C3—C4	1.371 (5)	C16—C17	1.372 (4)
C3—H3	0.9300	C16—H16	0.9300
C4—C5	1.376 (4)	C17—C18	1.366 (4)
C4—H4	0.9300	C17—N1	1.463 (4)
C5—N3	1.345 (4)	C18—H18	0.9300
C5—C6	1.471 (4)	N1—O5	1.205 (4)
C6—N2	1.331 (4)	N1—O6	1.224 (3)
C6—C10	1.382 (4)	O1W—H1W	0.81 (3)
C7—N2	1.326 (4)	O1W—H2W	0.82 (4)
C7—C8	1.369 (5)	O2W—H3W	0.82 (3)
C7—H7	0.9300	O2W—H4W	0.82 (4)
C8—C9	1.371 (6)	O3W—H5W	0.82 (4)
C8—H8	0.9300	O3W—H6W	0.82 (3)
C9—C10	1.361 (5)	O4W—H7W	0.81 (4)
C9—H9	0.9300	O4W—H8W	0.81 (3)
C10—H10	0.9300		
			
O1—Ni1—O2W	88.23 (9)	O2—C11—O1	125.6 (3)
O1—Ni1—O1W	89.39 (9)	O2—C11—C12	117.3 (3)
O2W—Ni1—O1W	173.81 (9)	O1—C11—C12	117.1 (3)
O1—Ni1—N2	94.35 (9)	C13—C12—C18	120.0 (3)
O2W—Ni1—N2	89.63 (10)	C13—C12—C11	120.1 (3)
O1W—Ni1—N2	96.25 (10)	C18—C12—C11	119.9 (3)
O1—Ni1—N3	170.92 (9)	C14—C13—C12	121.1 (3)
O2W—Ni1—N3	89.18 (9)	C14—C13—H13	119.5
O1W—Ni1—N3	94.02 (9)	C12—C13—H13	119.5
N2—Ni1—N3	76.92 (9)	C13—C14—C16	119.1 (3)
O1—Ni1—O3W	93.00 (9)	C13—C14—C15	119.5 (3)
O2W—Ni1—O3W	88.23 (10)	C16—C14—C15	121.3 (3)
O1W—Ni1—O3W	86.19 (10)	O3—C15—O4	124.8 (3)
N2—Ni1—O3W	172.27 (9)	O3—C15—C14	118.2 (2)
N3—Ni1—O3W	95.62 (9)	O4—C15—C14	117.1 (3)
N3—C1—C2	122.5 (3)	C17—C16—C14	119.0 (3)
N3—C1—H1	118.8	C17—C16—H16	120.5
C2—C1—H1	118.8	C14—C16—H16	120.5
C1—C2—C3	118.7 (3)	C18—C17—C16	122.9 (3)
C1—C2—H2	120.7	C18—C17—N1	118.6 (3)
C3—C2—H2	120.6	C16—C17—N1	118.5 (3)
C2—C3—C4	120.0 (3)	C17—C18—C12	117.8 (3)
C2—C3—H3	120.0	C17—C18—H18	121.1
C4—C3—H3	120.0	C12—C18—H18	121.1
C3—C4—C5	118.5 (3)	O5—N1—O6	123.2 (3)
C3—C4—H4	120.8	O5—N1—C17	118.1 (3)
C5—C4—H4	120.8	O6—N1—C17	118.7 (3)
N3—C5—C4	121.7 (3)	C7—N2—C6	118.3 (3)
N3—C5—C6	115.5 (2)	C7—N2—Ni1	125.9 (2)
C4—C5—C6	122.7 (3)	C6—N2—Ni1	115.4 (2)
N2—C6—C10	121.4 (3)	C1—N3—C5	118.7 (3)
N2—C6—C5	114.9 (3)	C1—N3—Ni1	126.6 (2)
C10—C6—C5	123.5 (3)	C5—N3—Ni1	114.67 (18)
N2—C7—C8	123.3 (3)	C11—O1—Ni1	125.67 (19)
N2—C7—H7	118.4	Ni1—O1W—H1W	105 (4)
C8—C7—H7	118.4	Ni1—O1W—H2W	113 (4)
C9—C8—C7	118.3 (3)	H1W—O1W—H2W	116 (3)
C9—C8—H8	120.8	Ni1—O2W—H3W	109 (3)
C7—C8—H8	120.8	Ni1—O2W—H4W	116 (4)
C10—C9—C8	119.0 (3)	H3W—O2W—H4W	113 (3)
C10—C9—H9	120.5	Ni1—O3W—H5W	109 (3)
C8—C9—H9	120.5	Ni1—O3W—H6W	120 (4)
C9—C10—C6	119.6 (3)	H5W—O3W—H6W	111 (3)
C9—C10—H10	120.2	H7W—O4W—H8W	116 (3)
C6—C10—H10	120.2		

### Hydrogen-bond geometry (Å, °)

**Table:**

*D*—H···*A*	*D*—H	H···*A*	*D*···*A*	*D*—H···*A*
O1W—H1W···O2	0.81 (3)	2.00 (3)	2.727 (3)	150 (5)
O1W—H2W···O4^i^	0.82 (4)	1.96 (5)	2.715 (3)	154 (4)
O2W—H3W···O4W^ii^	0.82 (3)	1.82 (4)	2.614 (4)	166 (4)
O2W—H4W···O4^iii^	0.82 (4)	1.96 (4)	2.757 (3)	164 (4)
O3W—H5W···O4W^iv^	0.82 (4)	2.42 (2)	3.185 (7)	156 (5)
O3W—H6W···O4^v^	0.82 (3)	1.97 (2)	2.735 (3)	155 (4)
O4W—H7W···O2^vi^	0.81 (4)	1.91 (2)	2.697 (4)	161 (5)
O4W—H8W···O3^vii^	0.81 (3)	1.98 (4)	2.639 (4)	138 (5)

Symmetry codes: (i) *x*, *y*−1, *z*; (ii) −*x*+2, −*y*+1, −*z*+1; (iii) *x*+1, *y*−1, *z*; (iv) *x*, *y*−1, *z*+1; (v) −*x*+1, −*y*+1, −*z*+2; (vi) −*x*+1, −*y*+1, −*z*+1; (vii) *x*+1, *y*, *z*−1.
